# N-acetylcysteine Restored Heart Rate Variability and Prevented Serious Adverse Events in Transfusion-dependent Thalassemia Patients: a Double-blind Single Center Randomized Controlled Trial

**DOI:** 10.7150/ijms.45795

**Published:** 2020-05-18

**Authors:** Sintip Pattanakuhar, Arintaya Phrommintikul, Adisak Tantiworawit, Somdet Srichairattanakool, Siriporn C. Chattipakorn, Nipon Chattipakorn

**Affiliations:** 1 Cardiac Electrophysiology Research and Training Center, Faculty of Medicine, Chiang Mai University, Chiang Mai, 50200, Thailand; 2 Department of Rehabilitation Medicine, Faculty of Medicine, Chiang Mai University, Chiang Mai, 50200, Thailand; 3 Division of Cardiology, Department of Medicine, Faculty of Medicine, Chiang Mai University, Chiang Mai, 50200, Thailand; 4 Division of Hematology, Department of Medicine, Faculty of Medicine, Chiang Mai University, Chiang Mai, 50200, Thailand; 5 Department of Biochemistry, Faculty of Medicine, Chiang Mai University, Chiang Mai, 50200, Thailand; 6 Center of Excellence in Cardiac Electrophysiology Research, Chiang Mai University, Chiang Mai, 50200, Thailand; 7 Cardiac Electrophysiology Unit, Department of Physiology, Faculty of Medicine, Chiang Mai University, Chiang Mai, 50200, Thailand

**Keywords:** transfusion dependent thalassemia, N-acetylcysteine, heart rate variability, oxidative stress

## Abstract

Regular blood transfusions in transfusion-dependent thalassemia (TDT) patients can lead to iron overload, causing oxidative stress and sympathovagal imbalance, resulting in increased cardiac complications. We hypothesized that administrating of N-acetylcysteine (NAC) prevents serious adverse events including cardiac complications in TDT patients by reducing systemic oxidative stress and balancing cardiac sympathovagal control. This study was double-blind, randomized control trial, investigating in 59 Thai TDT patients. After randomization, the participants were divided into two groups. The control group received standard care of TDT patient plus placebo, whereas the intervention group received 600 mg of NAC orally for six months. Serum 8-isoprostane, TNF-alpha, IL-10, 24-hour ECG monitoring, echocardiograms and the incidence of thalassemia-related complications were collected. At baseline, no significant difference in any parameters between the control and the intervention groups. At the end of intervention, the incidence of serious adverse events (i.e. infection, worsening thalassemia) was significantly higher in the control group when compared with the intervention group (24.1% vs. 3.3%, p=0.019) (Chi-square test; absolute risk reduction=20.8%, number needed to treat=4.8). The control group also had significantly lower time-dependent HRV parameters, compared with the intervention group (p=0.025 and 0.030, independent t-test). Treatment with NAC restored HRV and reduced serious adverse event in TDT patients, however, no difference in cardiac complications could be demonstrated. NAC could prevent serious adverse events in TDT patients. The proposed mechanism might be the balancing of sympathovagal control.

## Introduction

Thalassemia is a hereditary hematologic disorder caused by abnormal production of the globin chains of hemoglobin. Transfusion-dependent thalassemia (TDT) is the form of thalassemia in which regular red blood cell transfusions are necessary for survival of the patients 1. However, long-term red blood cell transfusions cause systemic iron overload, resulting in deposition and damages in iron-sensitive vital organs including the heart 2. Evidence shows that the leading cause of death in TDT patients is iron overload cardiomyopathy 3. Previous study demonstrated that early stage of iron overload cardiomyopathy could be associated with cardiac autonomic dysfunction, indicated by depressed heart rate variability (HRV) 4, 5. The second leading cause of death of TDT patients is infections 6. Evidence demonstrated that TDT patients had an immunocompromised status, regardless the history of splenectomy 6. Therefore, to reduce the complications of thalassemia, developing preventive interventions of iron overload cardiomyopathy as well as infectious conditions are in focus.

Evidence demonstrated that the mechanism of thalassemia-induced cardiac disorders was related to excessive oxidative stress 7. In thalassemia patients, systemic iron overload from excessive intestinal iron absorption and transfusional siderosis could induce Haber-Weiss and Fenton reaction, leading to cellular injury from lipid peroxidation, and causing damages to cellular proteins as well as nucleic acids, resulting in mitochondrial dysfunction, and apoptosis 8-10. NAC is a powerful antioxidant which is a precursor of L-cysteine which can instigate an increase in glutathione synthesis 11. Our previous studies using an iron overloaded model in mice and rats demonstrated that NAC attenuated oxidative stress in both peripheral blood as well as in heart tissues, resulting in protective effects against both cardiac dysfunctions evaluated by HRV and echocardiography 12-14. However, the effect of NAC on preventing cardiac and infectious complications in a clinical setting has not been investigated. In this study, we tested the hypothesis that in TDT patients, treatment with NAC reduces oxidative stress resulting in a prevention of complications and restore cardiac autonomic function, as indicated by lower complication rate and lower HRV depression, when compared with no treatment.

## Material and Methods

### Study protocol

The study protocol was approved by the Institutional Ethics Committee of the Faculty of Medicine, Chiang Mai University, Chiang Mai, Thailand and were in accordance with the 1964 Helsinki declaration and its later amendments or comparable ethical standards. The Thai Clinical Trials Registry number of this study was TCTR20160725002. The inclusion criteria included patients being in the age range from 18 to 50 years old and diagnosed with TDT as indicated by having regular red blood cell transfusions at least once a month after diagnosis**.** Patients with apparent heart diseases, diabetes mellitus, atrial flutter, atrial fibrillation, thyrotoxicosis, pacemaker installation, or had been taking any medications, which affected autonomic function including contraceptive, tricyclic antidepressant, anticholinergic agents, amphotericin B, calcium channel blocker, beta-blocker, antiarrhythmic, and centrally acting antihypertensive agents, were excluded prior to randomization. Medical records of the patients were reviewed to amass epidemiological data, transfusion history, and comorbidities**.** After giving informed consents, patients were randomized by the computerized program into two groups, which were the control and the NAC group. The results of randomizations were concealed in the envelopes and blind to the patients and the assessors until the end of the study. The control group received the standard treatment, including a regular one-month follow up with an outpatient blood transfusion when indicated (Hb < 7 mg/dl) and iron chelating agent (500 mg of deferiprone three time a day) plus a placebo. The placebo was a 600 mg-tablet of Maize starch administrating daily. The NAC group received oral 600 mg of NAC daily in addition to the standard treatment. The treatment was continued for six months following randomization. Routine laboratory tests, echocardiography, 24**-**h Holter ECG recording for HRV analysis and CMR T2* were investigated and analyzed two times, at baseline and at six months after randomization. Plasma 8-isoprostane (Abcam®, ab175819, Cambridge, UK), serum tumor necrotic factor alpha (TNF-α) (Abcam®, ab193687, Cambridge, UK), and serum interleukin-10 (IL-10) (Biosource International, Inc. Camarillo, CA, USA) were measured using enzyme-linked immunosorbent (ELISA) method 15, 16. Serum ferritin and plasma NTBI were determined using standard techniques as described previously 17, 18. Serious adverse events, as indicated by death, sepsis and worsening thalassemia (receiving more than two transfusions per month) in each patient, were identified and recorded. Non-serious adverse event was also identified and recorded.

### Echocardiographic studies

Standard two**-**dimensional and Doppler echocardiography was performed at rest in all enrolled patients with Philips iE33 (Philips Healthcare, Bothell, WA, USA)**.** Left ventricular end**-**diastolic dimension (LVEDD) and left ventricular end**-**systolic dimension (LVESD) were measured, and left ventricular ejection fraction (LVEF), stroke volume, cardiac output, and fractional shortening (FS) were determined based on the calculation of LV volumes by the method of discs, following the recommendations by the American Society of Echocardiography (ASE), and using apical two**-** and four**-** chamber views to measure cardiac function 19, 20**.** Impaired systolic LV dysfunction was determined by LVEF<55%, which was the specific value described in TDT patients 20**.** Diastolic function was evaluated from mitral inflow velocities (E**-**wave, A wave, E/A ratio) and deceleration time (DT) using pulsed wave (PW) Doppler in the apical four**-**chamber view**.** Diastolic function was investigated using E/A ratio according to the current guidelines 21**.**

### Heart rate variability measurement

HRV was evaluated using the SEER Light Holter system (GE Healthcare, Milwaukee, WI, USA)**.** An ECG was continuously recorded for 24 h**.** Prior to the HRV measurement, either exercise or caffeine consumption was prohibited in all participants. The computer software MARS software version 7, GE Healthcare, Milwaukee, WI, USA was used to scan for rhythm disturbance and to detect and label each QRS complex**.** Excessive noise and artifacts were noted, and ectopy was quantified**.** The time**-**domain analyses including average heart rate, average R**-**R intervals (NN), standard deviation of the R-R intervals over a 24-h period (SDNN), standard deviation of all 5**-**min mean R-R intervals (SDANN), average standard deviation of all 5-min R-R intervals (ASDNN), the percentage of R-R intervals with more than 50-ms variation (pNN50), and the square root of mean squared differences of successive R**-**R intervals (rMSSD). The frequency-domain analyses were determined with the same analytical software using Fast-Fourier transform analysis. The obtained frequency**-**domain indices were the total power (0-0.4 Hz), high-frequency power (HF, 0.15-0.4 Hz) spectral density, low-frequency power (LF, 0.04-0.15 Hz) spectral density, and very-low-frequency power (0.003-0.04 Hz) spectral density. These power spectral densities were described in absolute units (ms^2^). The designated physician, who operated and fitted the Holter monitor to the patients, was blinded to the patient information. The subjects were fitted with the Holter monitor after blood sample collection at the Holter unit of the Faculty of Medicine, Chiang Mai University. In the present study, none of the patients included in the study took medication such as beta**-**blockers, calcium channel blockers, statins, and ACE**-**inhibitors, which could affect the HRV 22.

### Magnetic resonance imaging

Magnetic resonance acquisition was performed using a 1.5T MR scanner (GE, HDxt, Milwaukee, WI, USA), with an 8**-**channel body array. For the measurements of myocardial iron overload, we used a T2 gradient-echo multi**-**echo sequence (25-degree flip angle, matrix: 192 x 128 pixels, 38 x 28.5 cm field of view,125-kHz bandwidth, 10.0**-**mm slice thickness, 1 number of excitations, 4 views per segment, 18.5-ms repetition time). A single mid-ventricular short-axis view of the left ventricle was acquired at eight echo times (TEs) (1.7 ms, which increased in 2.0-2.1 ms increments) in a single end**-**expiratory breath**-**hold. CMR T2* analysis was performed on a workstation (Advantage Window 4.6, GE healthcare) using commercial analysis software (StarMap 4.0, GE Healthcare). The values 14**-**20 ms were regarded as mild iron overload, values between 10 and 14 ms were regarded as moderate iron overload, and a T2* less than 10 ms was regarded as severe iron overload 23.

### Statistical analysis

Categorical variables were described using percentages of its frequency**.** Normally distributed numerical variables were presented using arithmetic means and standard deviations (SD)**.** Non-normally distributed variables were modified by taking a natural logarithm, and then presented using geometric mean and SD**.** Differences between the control and the NAC group were compared using the independent student t**-**test, Mann-Whitney U test or chi-square test, depending on the type of the parameters. The intention-to-treat analysis was used in this study. Statistical analyses were performed using SPSS version 22**.**0 for Windows (SPSS Inc**.**, Chicago, IL, USA)**.** A p-value of less than 0**.**05 was considered statistically significant**.**

## Results

### Baseline parameters

**Figure 1:**
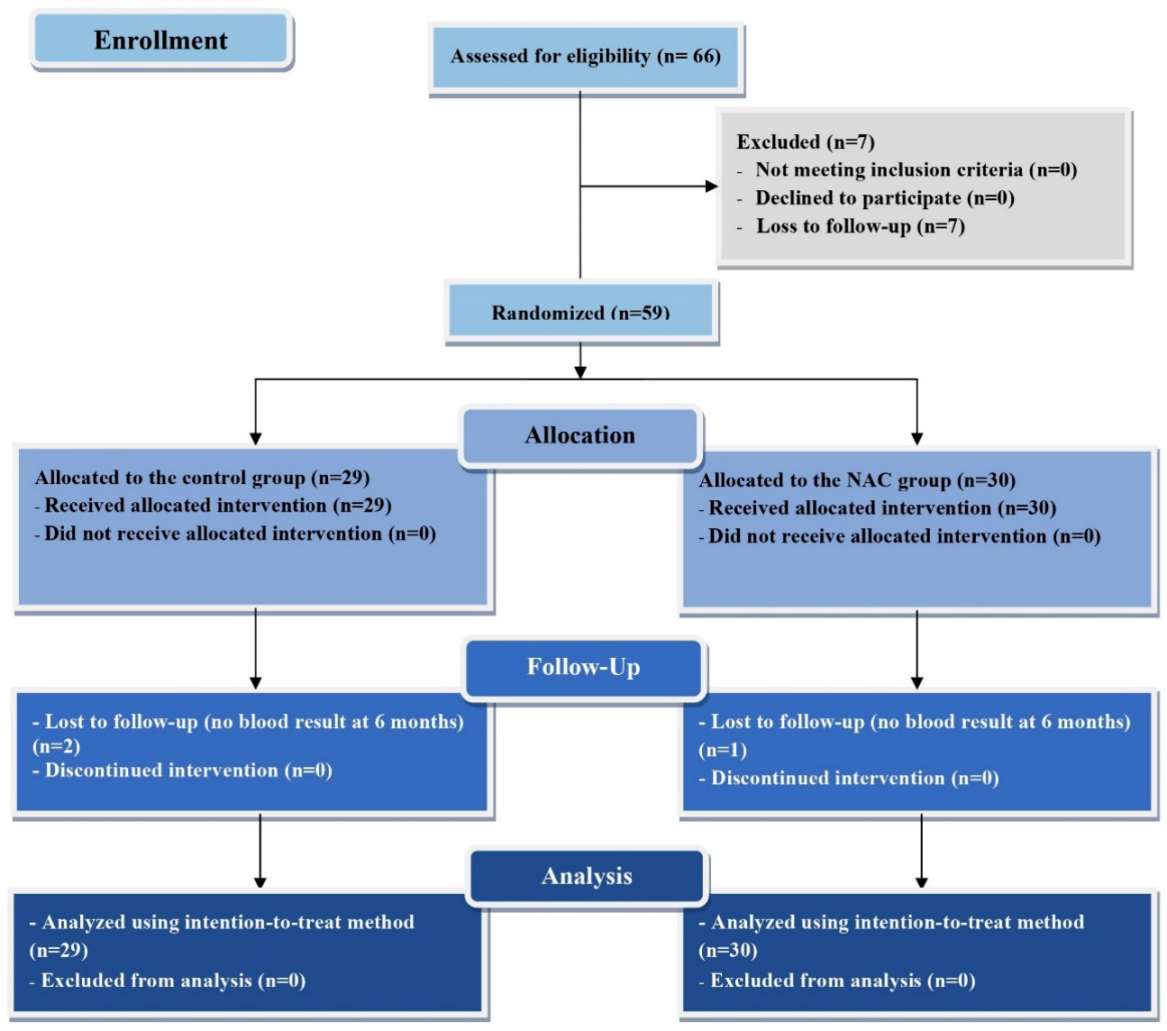
The CONSORT diagram for representing the protocol of this study

Sixty-six TDT patients were enrolled to the study. Seven patients lost their follow-up before randomization. Twenty-nine patients were randomized into the control group and thirty patients were randomized into the NAC group. Two patients in the control group and one patient in the NAC group were lost to follow up during the six-month treatment period. The demographic, clinical, biological, cardiac imaging and HRV parameters at baseline of the study were presented in Table 1. No significant difference in all parameters was found between the control and the NAC group at baseline. The CONSORT diagram for representing the protocol of this study is as shown in Figure 1.

### The effect of NAC on preventing serious adverse events

Table 2 demonstrated the differences in each parameter between the control and the NAC group after six-month treatment of NAC. Since we used the intention to treat analysis, the missing data were estimated by using the worst-case imputation for categorical parameters and using the last observation carries forward imputation in continuous parameters. Five patients in the control group developed serious adverse events. One patient died from septic shock. Two patients had sepsis from acute cholecystitis. One patient had sepsis from acute pyelonephritis. One patient was diagnosed with worsening thalassemia, as defined by receiving more than two transfusions per month. All serious adverse events were detected at the sixth month of the study. No patient from the NAC group developed a major complication. Using *Chi*-square test, it was demonstrated that the rate of serious adverse events was higher in the control group when compared with the NAC group (24.1% vs. 3.3%, p=0.019). The absolute risk reduction between the control group and the NAC group was 20.8 and the number needed to treat was 4.8. We also performed the per-protocol analysis and found a statistical significance in serious adverse event rate between the control and the NAC group (18.5% vs. 0%, p=0.021). No non-serious adverse event was found in both groups.

### The effect of NAC on HRV, cardiac imaging and HRV biological parameters

At six-month time point, by using intention-to-treat analysis and the last observation carries forward imputation for missing data, we found that the control group had significantly lower SDNN and SDANN parameters (p=0.025 and 0.030, independent *t*-test, respectively), indicating more depressed HRV, when compared with the NAC-treated group (Table 2). Moreover, these significant differences were also found when using per-protocol analysis (p=0.041 for SDNN and p=0.044 for SDANN). There was no difference in the other time-domain parameters, as well as all the frequency-domain HRV parameters, between the control and the NAC group. There was no significant difference in the echocardiographic parameters including the systolic function measured by LVEF, diastolic function measured by E/A ratio, as well as cardiac iron status measured by CMR T2* between the control and the NAC group at six-month time points (Table 2). In this study, no patient was diagnosed with iron overload cardiomyopathy by the criteria of CMR T2* < 10. When switching the criteria of CMR T2* to < 20, four patients from the control group and two patients from the NAC group were potentially diagnosed with impending iron overload cardiomyopathy after the six-month intervention. However, there was still no significant difference between the two groups. Focusing on the biological parameters, we found that there was no significant difference in oxidative stress marker plasma 8-isoprostane, pro-inflammatory cytokine serum TNF-α, anti-inflammatory cytokine serum IL-10, serum ferritin and plasma NTBI between the control and the NAC group at six-month month (Table 2).

## Discussion

The major findings of this study were that TDT patients treated with NAC had significantly lower rate of serious adverse event with the absolute risk reduction of 20.8% and the number needed to treat of 4.8, as well as lower HRV depression when compared with the control group. There was no significant difference in 8-isoprostane, TNF-alpha and IL-10 level, and the incidence of cardiac complications between the NAC treatment and the control group. These results confirmed our hypothesis that NAC treatment promotes the benefits to TDT patients via balancing the sympathovagal activity. However, there are many interesting points to be discussed regarding these results.

The most common cause of death of TDT patients is heart failure from iron overload cardiomyopathy 3. However, in this study, none of the patient was diagnosed with iron overload cardiomyopathy nor heart failure. The explanation for the absence of iron overload cardiomyopathy in this study could be due to several reasons including the relatively short follow-up period, the recruited low-risk patients with a normal cardiac T2*, and the concomitant early administration of iron chelator. This result may reflect an effective strategy of early iron chelator administration in order to prevent the cardiac iron overload status in TDT patients 24. The second most common and fatal complications in TDT patients is infections 6. Worsened thalassemia as indicated by having more often blood transfusion is usually also resulted from infections 6. Infections are affected by autonomic function since the balance between sympathetic and parasympathetic nervous systems plays an important role in creating proper immune responses 25, 26. It was hypothesized that extremely low parasympathetic to sympathetic ratio is associated with poor immune responses, whereas extremely high parasympathetic to sympathetic ratio is associated with excessive immune reactions 26. We proposed that the effect of NAC on preventing serious adverse events in TDT patients might result from its effect on balancing sympathovagal activity. This result was relevant to the result of HRV, which demonstrated the attenuation of HRV depression after NAC treatment.

Previous studies have demonstrated that patients with thalassemia developed cardiac autonomic dysfunction 27. Our previous studies also demonstrated that the cardiac autonomic dysfunction detected in thalassemia patients was positively correlated with cardiac iron overload status 17, 28, 29. In adults with TDT and non-TDT, LF/HF ratio parameter in HRV had a significantly positive correlation with CMR T2* value 28, 29. In children with TDT, SDNN, SDANN, ASDNN, LF and HF parameters of HRV were positively correlated with CMR T2* value 17. It was also suggested that in TDT, cardiac autonomic dysfunction detected by HRV occurs earlier than cardiac iron overload detected by CMR T2* 29. In addition, cardiac contractile dysfunction is proposed to occur after cardiac iron overload 29. This might be an explanation that in this study, NAC treatment could prevent cardiac autonomic dysfunction but has no effect on cardiac iron overload status or cardiac contractile function, since these later two complications had not been occurred in our patients.

The mechanisms involved in the depression of HRV in TDT patients could mainly be mediated by oxidative stress. In thalassemia patients, systemic iron overload occurred via two mechanisms, from excessive intestinal iron absorption and from transfusional siderosis 8. Excessive iron acquisition causes saturation of transferrin-binding capacity, resulting in the presence of plasma NTBI 2. NTBI can deposit in various tissues, such as the reticulo-endothelial system of the spleen, liver, bone marrow and in advanced cases, in the cardiac myocytes 30, 31. Deposition of NTBI in those tissues causes oxidative stress by enhancing reactive oxygen species (ROS) via the Haber-Weiss and Fenton reaction, leading to cellular injury from lipid peroxidation, damage to cellular proteins as well as nucleic acids, mitochondrial dysfunction, and apoptosis 10, 32. Previous studies have shown that the oxidative stress from many pathological conditions, including iron overload, can promote sympathovagal disturbance and cause significantly depressed HRV 13, 33, 34. Therefore, the depressed HRV observed in this study could be mainly caused by oxidative stress mechanisms.

NAC is known as a potent antioxidative agent. Many evidences demonstrated that NAC was an effective treatment of oxidative stress-mediated diseases 11. Our *in vivo* studies demonstrated that treatment with NAC could prevent oxidative stress, as indicated by a reduction in malondialdehyde (MDA) level, in plasma and cardiac tissues 12-14. However, the present study failed to demonstrate the effect of NAC on reducing oxidative stress since no significant difference in 8-isoprostane level between the NAC group and the control group. The inconsistent result in this study might be due to the time-dependent effect of red blood cells transfusion on oxidative stress in plasma of each patient. Previous *in vivo* evidence demonstrated that although standardly restored, red blood cells displayed significant changed in oxidative stress markers 35. Another clinical study comparing between pre- and 12 hours post-transfusion demonstrated that red blood cell transfusion was associated with increased oxidative damage markers 36. All patients in this study received at least one time of blood transfusion per month. In this study, no data regarding the time between blood collections and blood transfusions was reported. Therefore, the duration between red blood cells transfusion and blood collection for evaluating oxidative stress marker might be responsible for the insignificant result of 8-isoprostane in our study. In addition, the results of serum ferritin and plasma NTBI were not significantly different between the control and the NAC group. This result suggested that NAC treatment is proposed to prevent complications from oxidative stress, not to decrease the iron overload status, resulting in no effect on iron overload parameters such as ferritin and NTBI levels.

Regarding using NAC in thalassemia, the results of our study were compatible with the previous clinical study. For example, Ozdemir, et al. (2014) reported that NAC supplementation reduced oxidative stress measured by the oxidative stress index and decreased DNA damages in children with TDT 37. Yanpanitch, et al. (2015) also reported that treatment with antioxidant cocktails (NAC plus vitamin E or curcuminoids) decreased oxidative stress measured by red blood cell MDA level, increased hemoglobin concentration and reduced hypercoagulable state in β thalassemia/Hb E TDT patients 38. However, no data on clinical complication was reported in these two studies. To the best of our knowledge, our study is the first study demonstrating the effect of NAC on preventing complications in TDT patients.

Regarding limitations, the most important limitation of this study is a small sample size potentially causing inadequate statistical power to detect the difference of parameters between the two groups, which could also be responsible for the negative results of oxidative marker in this study. Future large randomized control trial calculated the sample size based on results of this study, and uniformly collecting plasma sample more than 12 hours after blood transfusion is needed to clarify the mechanism of the effect of NAC on preventing complications in TDT patients. One limitation in this study is that NT-pro-BNP was not determined in these patients. Future study needs to investigate this cardiac biomarker in addition to echocardiogram and cardiac MRI.

## Conclusion

Treatment with NAC could restore HRV and prevented serious adverse events in TDT patients, however, no difference in cardiac complications could be demonstrated. This result addresses the importance of using NAC in the routine treatment to improve cardiac autonomic function and decrease complications in TDT patients.

## Figures and Tables

**Table 1:** Baseline characteristic of the participants

Parameters	All patients (N = 59) Mean (SD)	NAC group (N = 30) Mean (SD)	Control group (N = 29) Mean (SD)	p-value
***Demographic data***				
**Age (years)**	27 (8)	28 (8)	27 (8)	0**.**515
**Sex (male/female) (%)**	39/61	34/66	44/56	0**.**446
**Number of transfusions in last 12 months (times)**	16 (5)	16 (5)	16 (5)	0**.**817
***Biochemical parameters***				
**Hb (g/dL)**	7**.**15 (1**.**1)	7**.**14 (1**.**21)	7**.**16 (0.97)	0**.**955
**Hct (%)**	23**.**47 (4.04)	23.33 (4.35)	23.61 (3.75)	0**.**794
**Geometric mean of serum ferritin (95% CI) (ng/dL)**	1554 (1278,1889)	1561 (1149,2120**)**	1546 (1194,2002)	0**.**961
**Plasma NTBI (µM)**	6**.**79 (1**.**95)	6**.**59 (2)	6**.**98 (1**.**92)	0**.**503
***Inflammatory and oxidative stress markers***				
**Serum TNF-alpha (ng/dL)**	16.67 (7.10)	17.14 (7.32)	16.16 (6.97)	0.612
**Geometric mean of Serum IL-10 (95% CI) (ng/dL)**	1.25 (0.96,1.61)	1.51 (1.10,2.08)	1.00 (0.66,1.53)	0.103
**Serum 8-isoprostane (ng/dL)**	17.99 (2.26)	18.02 (2.11)	17.95 (2.46)	0.912
***Cardiac imaging parameters***				
**LVEF (%)**	69 (4)	69 (6)	69 (4)	0**.**375
**LV diastolic volume (ml)**	87 (23)	85 (21)	87 (25)	0.952
**E/A ratio**	1**.**6 (0**.**6)	1**.**6 (0**.**4)	1**.**6 (0**.**7)	0**.**972
**TRV max**	254 (52)	261 (60)	247 (43)	0**.**533
CMR T2* (ms)	37.42 (13.3)	38.05 (13.93)	36.74 (12.81)	0**.**714
Impending cardiac iron overload^†^ (CMR T2* < 20 ms) (%)	5 (8**.**92)	3 (10.34)	2 (7.40)	0.701
***HRV-frequency domain***				
**VLF (ms^2^)**	19.32 (6.30)	19.63 (6.92)	18.98 (5.66)	0.703
**LF (ms^2^)**	11.37 (4.61)	11.85 (5.34)	10.84 (3.70)	0**.**416
**HF (ms^2^)**	8.03 (4.00)	8.47 (4.34)	7.56 (3.60)	0**.**396
**LF/HF ratio**	1**.**51 (0**.**37)	1**.**51 (0**.**42)	1**.**52 (0**.**32)	0**.**857
***HRV-time domain***				
**SDNN (ms)**	96.46 (27.73)	100.28 (29.75)	92.37 (25.30)	0**.**293
**SDANN (ms)**	88.77 (27.60)	92.00 (23.34)	84.59 (25.50)	0**.**278
**ASDNN (ms)**	35.55 (11.35)	36.69 (12.38)	34.33 (10.22)	0**.**446
**rMSSD (ms)**	20.09 (9.56)	21.52 (10.19)	18.56 (8.75)	0**.**250

All statistical analyses were performed by independent t-test except † by chi-square test * significant at p<0.05 TDT, transfusion-dependent thalassemia; NAC, N-acetylcysteine; SD, standard deviation; Hb, hemoglobin; Hct, hematocrit; TNF-α; tumor necrotic factor alpha; IL-10, interleukin-10; NTBI, non-transferrin-bound iron; LVEF, left ventricular ejection fraction; LV, left ventricle; TRV max, maximum velocity of tricuspid regurgitant flow; HRV, heart rate variability; LF, low frequency power; HF, high frequency power; LF/HF ratio, ratio of power in low/high frequency; SDNN, standard deviation of all normal sinus R-R intervals in the entire 24-h recording; SDANN, standard deviation of average of all normal sinus R-R intervals for all 5-min segments in the 24-h recordings; ASDNN, average of the standard deviations of all R-R intervals for all 5-min segments in the 24-h recordings; rMSSD, root mean square of the mean of the squared difference of two consecutive R-R intervals

**Table 2:** The effects of six-month N-acetylcysteine treatment on clinical, biological, cardiac imaging and heart rate variability parameters in TDT patients

Parameters	All patients (N = 59) Mean (SD)	NAC group (N = 30) Mean (SD)	Control group (N = 29) Mean (SD)	p-value
*Demographic data*				
Age (years)	28 (8)	29 (8)	28 (8)	0**.**513
N of patients who having serious adverse events (infection, sepsis, worsening thalassemia, death) at 6 months^†^	8	1	7	0**.**019^*^
Number of transfusions during intervention (times)	8 (3)	8 (3)	8 (3)	0**.**889
*Biochemical parameters at 6 months*				
Hb (g/dL)	7**.**42 (1**.**40)	7**.**44 (1**.**60)	7**.**39 (1.20)	0**.**893
Hct (**%**)	23**.**79 (4.29)	23.81 (4.56)	23.77 (4.08)	0**.**978
Geometric mean of serum ferritin (95**%** CI) (ng/dL)	1688 (1371,2078)	1603 (1126,2281)	1785 (1417,2248)	0**.**605
Geometric mean of plasma NTBI (µM)	2.38 (1**.**95,2.91)	2.56 (1.91,3.43)	2.21 (1**.**66,2.95)	0**.**477
*Inflammatory and oxidative stress markers at 6 months*				
Serum TNF-α (ng/dL)	12.38 (4.47)	12.11 (4.61)	12.54 (4.35)	0.725
Geometric mean of Serum IL-10 (95**%** CI) (ng/dL)	1.08 (0.80,1.61)	1.06 (0.67,1.67)	1.09 (0.72,1.65)	0.929
Serum 8-isoprostane (ng/dL)	21.01 (4.83)	20.34 (5.21)	21.74 (4.28)	0.279
*Cardiac imaging parameters at 6 months*				
LVEF (**%**)	69 (5)	69 (5)	69 (5)	0**.**514
LV diastolic volume (ml)	84 (19)	84 (18)	84 (21)	0.994
E/A ratio	1**.**7 (0**.**6)	1**.**7 (0**.**6)	1**.**6 (0**.**5)	0**.**177
TRV max	249 (46)	249 (49)	248 (43)	0**.**993
CMR T2* (ms)	34.14 (12.2)	33.69 (13.98)	34.63 (10.99)	0**.**774
Impending cardiac iron overload^†^ (CMR T2* < 20 ms) (%)	6 (10.71)	4 (13.79)	2 (7.40)	0.671
*HRV****-****frequency domain parameters at 6 months*				
VLF (ms^2^)	19.49 (6.40)	20.73 (6.98)	18.16 (5.54)	0.137
LF (ms^2^)	11.50 (5.07)	12.50 (5.85)	10.43 (3.90)	0**.**126
HF (ms^2^)	8.20 (4.62)	9.00 (5.04)	7.34 (4.04)	0**.**185
LF/HF ratio	1**.**52 (0**.**37)	1**.**50 (0**.**41)	1**.**54 (0**.**32)	0**.**781
*HRV****-****time domain parameters at 6 months*				
SDNN (ms)	104.4 (31.7)	113.28 (32.03)	95.39 (25.93)	0**.**025^*^
SDANN (ms)	97.6 (31.2)	105.65 (30.8)	88.82 (26.57)	0**.**030^*^
ASDNN (ms)	35.9 (12.1)	39.06 (12.58)	33.21 (10.42)	0**.**058
rMSSD (ms)	21.1 (10.7)	23.10 (10.20)	19.04 (10.36)	0**.**135

All statistical analyses were performed by independent t-test except † by chi-square test * significant at p<0.05 TDT, transfusion-dependent thalassemia; NAC, N-acetylcysteine; SD, standard deviation; Hb, hemoglobin; Hct, hematocrit; TNF-α; tumor necrotic factor alpha; IL-10, interleukin-10; NTBI, non-transferrin-bound iron; LVEF, left ventricular ejection fraction; LV, left ventricle; TRV max, maximum velocity of tricuspid regurgitant flow; HRV, heart rate variability; LF, low frequency power; HF, high frequency power; LF/HF ratio, ratio of power in low/high frequency; SDNN, standard deviation of all normal sinus R-R intervals in the entire 24-h recording; SDANN, standard deviation of average of all normal sinus R-R intervals for all 5-min segments in the 24-h recordings; ASDNN, average of the standard deviations of all R-R intervals for all 5-min segments in the 24-h recordings; rMSSD, root mean square of the mean of the squared difference of two consecutive R-R intervals
